# Magnetic Foreign Body Ingestion in Children: The Attractive Hazards

**DOI:** 10.1155/2019/3549242

**Published:** 2019-04-28

**Authors:** Anna Lin, Lawrence Chi Ngong Chan, Kam Lun Ellis Hon, Siu Yan Bess Tsui, Kristine Kit Yi Pang, Hon Ming Cheung, Alexander K. C. Leung

**Affiliations:** ^1^Department of Paediatrics, The Chinese University of Hong Kong, Hong Kong; ^2^Department of Surgery, The Chinese University of Hong Kong, Hong Kong; ^3^Department of Pediatrics, The University of Calgary, Calgary, Alberta, Canada

## Abstract

Foreign body ingestions are frequent in the childhood population. Most foreign bodies are passed spontaneously through the gastrointestinal tract. However, on occasion, they can also be a rare cause of morbidity and even mortality, such as in the case of multiple magnetic foreign body ingestion, which can cause injury via magnetic attraction through bowel walls. We present two cases of multiple magnetic foreign body ingestion, which to our knowledge are the first ones reported in Hong Kong. One patient presented with shock and intestinal necrosis requiring extensive intestinal resection, whereas the other patient had no gastrointestinal injury but surgical removal was deemed necessary.

## 1. Introduction

Foreign body (FB) ingestions are frequent in childhood accidents and injuries and occur most commonly in children between 6 months and 6 years of age [1–3]. Toys not only provide enjoyment but also tend to be inherently attractive to young children; they are also essential tools for a child's learning and development. However, toys are also a form of FB that is occasionally ingested by curious young children. The increasing popularity of magnets for refrigerators, magnetic jewelry, and magnetic toy building and sculpting sets has led to the wider availability of these magnetic objects and an increased incidence of magnetic FB ingestion, necessitating endoscopic or surgical interventions being reported in the literature in recent years [4–7]. The ingestion of magnetic objects poses a significant health risk to children, especially with multiple magnetic objects ingestion, as magnetic attraction through bowel walls can cause gastrointestinal injury such as mural pressure necrosis, bowel perforation, fistula formation, or intestinal obstruction [8]. The public should be aware of the danger associated with these magnetic objects.

## 2. Case Reports

### 2.1. Case 1

**Figure 1:**
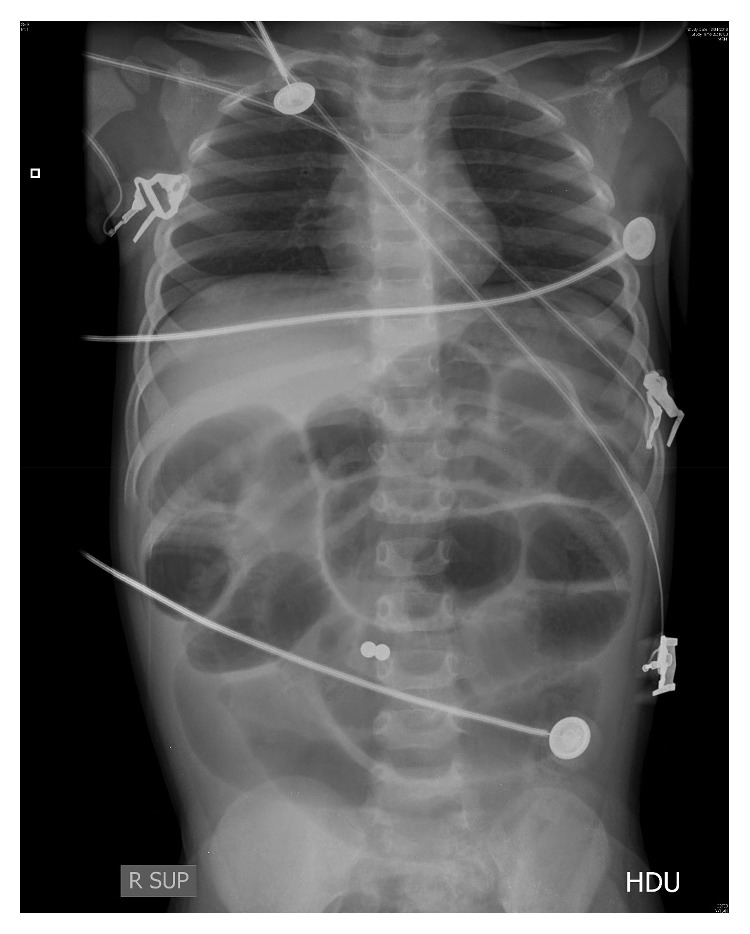
Diffuse bowel dilatation and two circular radiopaque opacities in the bowel suspicious of foreign body.

**Figure 2:**
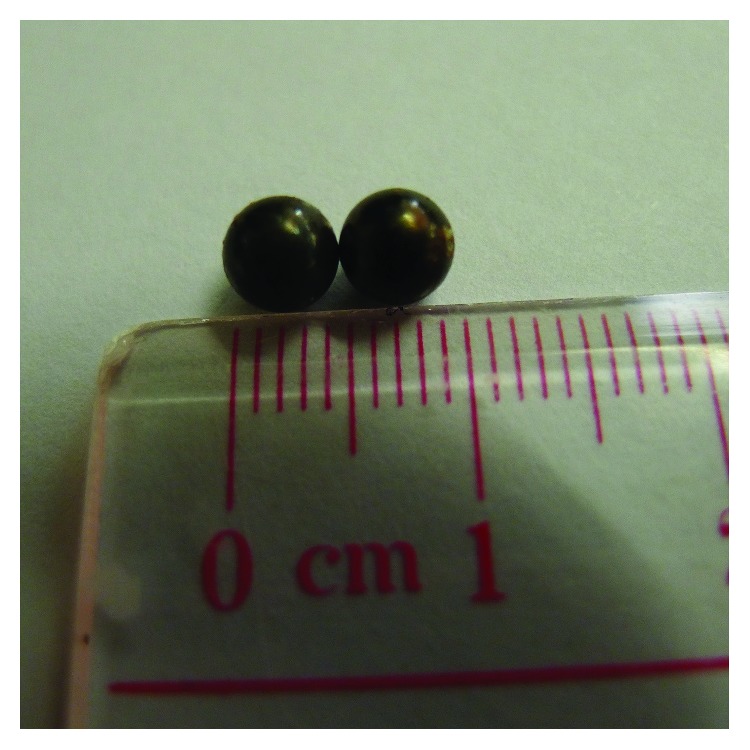
Two metallic beads were removed during surgery for gangrenous small bowel.

A 27-month-old previously healthy boy presented to the emergency department with repeated vomiting, sweating, generalized weakness, dizziness, anxiety, and reduced consciousness. He was found to be in shock with a heart rate of 200 beats per minute, respiratory rate of 49 breaths per minute, and blood pressure of 84/43 mmHg. The abdomen was soft but grossly distended with sluggish bowel sounds. Arterial blood gas revealed metabolic acidosis with a pH of 7.12 and base excess of −14. The arterial lactate level was 5 mmol/L. Initial abdominal radiography showed diffuse bowel dilatation but no apparent air-fluid level and two circular radiopaque opacities in the bowel suggestive of metallic foreign bodies (Figure 1). The patient was admitted to the paediatric intensive care unit for resuscitation. He was stabilized with intravenous fluids and ionotropic support. Emergency laparotomy revealed small bowel obstruction with extensive necrosis. Approximately 107 cm of gangrenous small bowel was resected, and end-to-end anastomosis was performed. Two magnetic beads sized 5 mm × 5 mm were found (Figure 2), one in the small bowel and the other in the right colon. The magnetic beads were removed. Postoperative recovery was uneventful. Retrospective questioning of the parents revealed no history suspicious of FB ingestion.

### 2.2. Case 2

**Figure 3:**
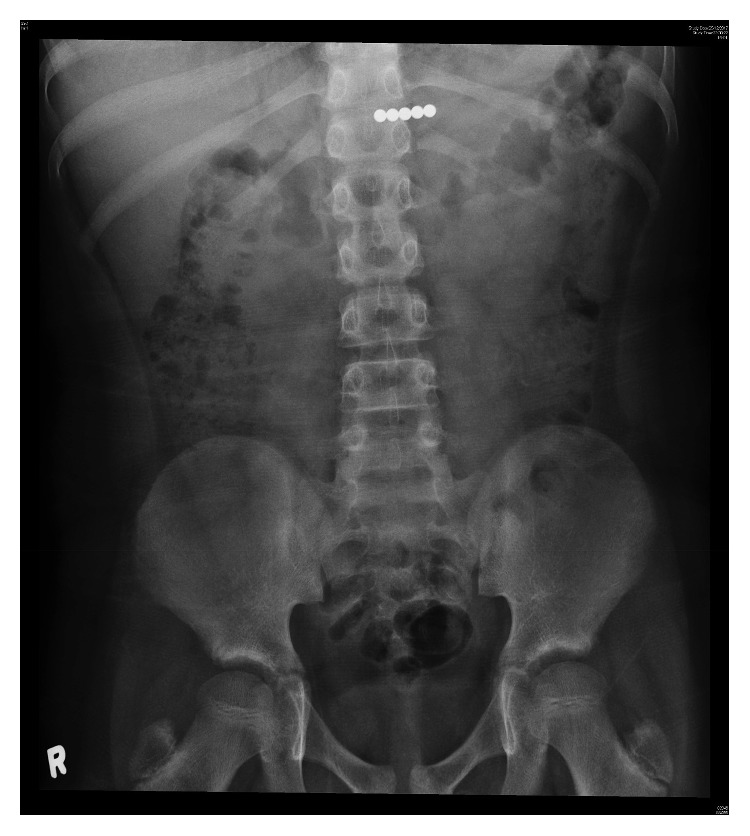
Five linear round radiopaque opacities in the epigastrium of an asymptomatic child.

A 9-year-old boy presented to the emergency department immediately after accidental ingestion of magnetic beads. The patient was asymptomatic and vital signs were stable. There were no signs of obstruction or perforation. Initial abdominal radiography showed five round radiopaque objects in the epigastrium (Figure 3). Aggressive management was employed in view of the multiplicity of the beads ingested and potential risk of serious complications. Emergency oesophagogastroduodenoscopy showed no foreign body up to the second part of the duodenum. The beads had moved further beyond the duodenum. Laparoscopy was then performed which revealed a string of five magnetic beads adhered to each other in the small bowel. The beads were removed via enterotomy. The patient remained asymptomatic and stable and made an uneventful recovery.

## 3. Discussion

The presentations and outcomes of these two children with multiple magnetic bead ingestion differed greatly. In the former, unwitnessed multiple magnetic bead ingestion led to a delayed presentation and complications of intestinal necrosis requiring extensive bowel resection, whereas the latter patient was brought to the emergency department immediately after accidental ingestion of multiple magnetic beads, which was likely a major factor for the better outcome.

In the former case, we postulate that the unusual mechanism of injury was due to strong magnetic forces, which adhered two different loops of bowel together during peristalsis. Despite the minimal contact surface areas between the two beads, these magnetic forces were potent enough to impair gut motility. Impaired peristalsis led to twisting, ischemia, and necrosis of the gut, resembling volvulus. The incident did not take place in the oesophagus or the stomach, which might indicate that the two beads were swallowed sequentially. Theoretically speaking, it would not have occurred if the two beads were stuck together in the early course of the transit in the gastrointestinal tract. The two beads were small and looked like candies. Curious children tend to put small items such as coins, marbles, toy parts, button batteries, bones, and pills in their mouths [9, 10]. It is the responsibility of parents and caregivers to be vigilant about possible ingestion of small items and toys in the vicinity of a child.

Based on these two cases, it follows that obstruction and ischemia in the lower intestinal tract following magnetic FB ingestion appear to be more severe and symptomatic compared to nonmagnetic FBs. This is especially true for high-powered magnets made of neodymium [9]. Clinicians should be aware that the later the initiation of treatment, the more severe the sequelae may be.

A systematic review of gastrointestinal injury caused by magnetic FB ingestions in children and adolescents found most children were younger than 6 years, magnetic FBs ingested were mainly toys, the number of FBs ranged from 2 to 100, and the majority of patients were previously healthy [4]. Delayed diagnosis and treatment existed in all of the patients to varying extents. Those who underwent exploratory laparotomy showed a wide range of bowel damage was possible, including perforation and intestinal fistula. Intestinal damage was the most common injury, followed by entero-colonic fistula. In that series, most patients required bowel resection with anastomosis or fistula repair except for two children who were managed by endoscopic removal of the FB. In a study of 72 children (mean age 6.4 years) with rare-earth magnet ingestion, the clinical outcome was specified in 93.1% (67/72) of these patients [11]. Of these 67 children, 22 (32.8%) had no adverse effects. Intervention was reported in 91.7% (66/72) of cases. Surgical intervention was required in 46 children (69.7%). Endoscopic removal was performed in 7.6% of cases. The remaining 21.2% of patients were treated conservatively with the magnets passing naturally without intervention. The number of magnets ingested ranged from 1 to 40. Ingestion of 2 to 4 magnets comprised 44.4% of the cases [11]. In general, ingesting more than one magnet can potentially lead to severe gastrointestinal injury, such as mural pressure necrosis, bowel perforation, peritonitis, intra-abdominal sepsis, fistula formation, volvulus, intestinal obstruction, ischemia, and death [5,7,12–14]. For symptomatic patients, multiple magnetic FB ingestion, or when the magnetic FB is in the stomach or the oesophagus, early surgical intervention can prevent significant morbidity and mortality [12, 15]. As with our cases, clinical vigilance should be exercised and early surgical consultation with an aggressive surgical approach is recommended. If the magnetic FB is in the oesophagus, stomach, or proximal small bowel, endoscopy should be performed to retrieve the object and to examine possible damage that might have been caused [9]. For asymptomatic patients, a conservative approach should be considered with serial abdominal radiography to monitor whether the magnetic beads remain in the same location and to wait for spontaneous passage of the magnetic FB. This is especially so if only one magnetic FB is ingested [12]. Failure of movement of the magnetic FB or development of gastrointestinal symptoms prompts reconsideration of endoscopic or surgical intervention [12]. Suffice to say that in a significant number of patients with magnetic FB ingestion, the magnetic objects pass through the gastrointestinal tract spontaneously without complications [16]. However, two or more magnetic FBs or a single magnet coingested with other metallic objects can attract each other in the gastrointestinal tract. This may cause ischemia of the bowel wall and pressure necrosis of the bowel and warrant early surgical intervention [14, 16, 17].

Parents should be warned of the danger of toys that contain metals or magnets. It is not easy to distinguish whether the ingested FB is metallic or magnetic [3]. It is recommended that young and at-risk children should not have access to toys or objects that contain small magnets or metals [18]. In this regard, the US Consumer Product Safety Commission has mandated magnet toys not to be sold to individuals under the age of 14 years [14]. Improved regulation and magnet safety standards are needed. Ideally, magnets should be large enough to decrease the chance of being ingested and the magnetic force be lowered to a flux index of 50 kG^2^·mm^2^, which is approximately 37 times weaker than some magnetic toys in circulation [12].

## 4. Conclusion

Parents and caregivers should remove high-powered small magnets from the reach of children. Physicians must be vigilant on reviewing the radiology of a child presenting with respiratory or gastrointestinal symptomatology and not to assume radiopaque objects are extracorporeal. A high index of suspicion is necessary in patients presenting with unexplained gastrointestinal symptoms, and aggressive and early removal is warranted in cases of multiple magnetic FB ingestion to reduce potential morbidity and mortality.
